# P-2345. Recent Incidence Rate of Herpes Zoster Among Immunocompetent Adults Aged 18 and Older in the United States

**DOI:** 10.1093/ofid/ofae631.2497

**Published:** 2025-01-29

**Authors:** Rachel A Cohen, Yves Brabant, Driss Oraichi, Agnes Mwakingwe-Omar, Bruno Anspach, Desmond Curran, Mitra Yousefi, Huifeng Yun

**Affiliations:** GSK, Rockville, Maryland; GSK, Rockville, Maryland; GSK, Rockville, Maryland; GSK, Rockville, MD, USA, Rockville, Maryland; GSK, Rockville, Maryland; GSK, Rockville, Maryland; GSK, Rockville, Maryland; GSK, Rockville, Maryland

## Abstract

**Background:**

There is a lack of current herpes zoster (HZ) incidence data among younger age groups of immunocompetent adults in the United States (US), with rigorous exclusion criteria for potential immunocompromising (IC) or chronic conditions. Here we assess not only incidence but also HZ severity across age groups.

**Table 1.**
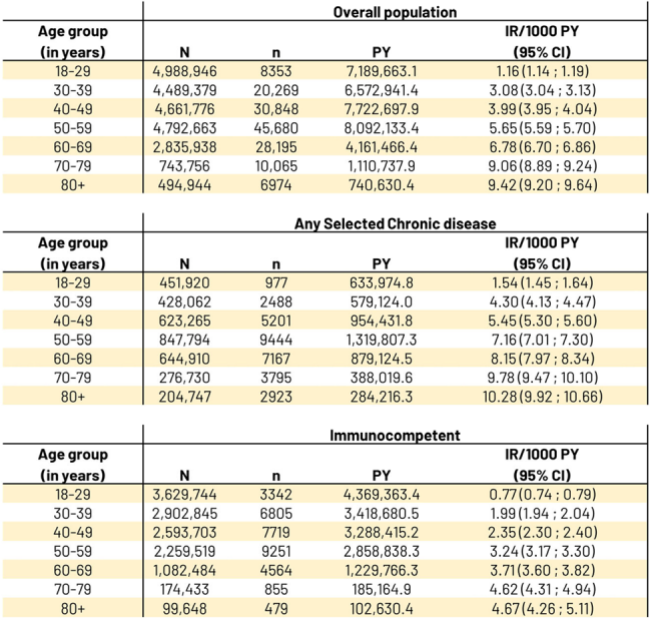
US HZ IR/1000 PY by age groups in: overall population, selected chronic disease, and immunocompetent participants between January 1, 2017 to March 31, 2022

- IR: Incidence Rate. PY: Person Years at risk. CI: Confidence Interval. US: United States.

- N=number of people at risk at corresponding age group (time-varying) during their follow up period.

- n=number of people with HZ among N.

**Methods:**

Our retrospective cohort study used 2015-2022 Merative MarketScan database to estimate age-specific incidence rate (IR) of HZ (ICD-10 code B02.xx and antiviral ± 7 days) among ≥18 years of age (YOA), continuously enrolled for 15 months, with no prior diagnosis of or vaccination against HZ: 1- overall; 2- with selected chronic disease [i.e., diabetes, asthma, COPD, CKD, depression, stress, and/or trauma] and no IC/Auto-immune disease (AID); 3- Immunocompetent [no IC/AID conditions/medications, no chronic conditions]. Index date was 1st day of 16th month of continuous enrollment. Individuals were assigned to ∼10 year age groups, reassessed annually. Censoring occurred at first HZ, first zoster vaccine, or loss of coverage. HZ post-herpetic neuralgia (PHN) %, HZ hospitalization % were calculated. Sensitivity analysis was conducted using only one diagnosis code.

**Table 2.**
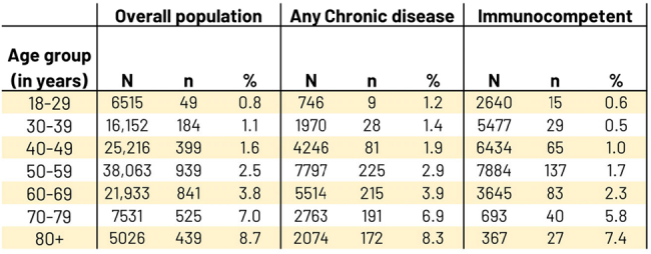
Number (%) of HZ cases that developed PHN by age groups in: overall population, selected chronic disease, and immunocompetent participants

- N=number of people with HZ in age group.

- n=number of people from N who have PHN with at least 180 days of continuous enrollment following HZ.

- %=n/N*100.

- Note: Age is computed at HZ event date.

**Results:**

20,673,677 participants met inclusion criteria overall. 15.3% had ≥1 selected chronic disease, 57.2% were immunocompetent. Overall HZ IR increased with age: 1.16, 3.08, 3.99, and 5.65/1000 Person-Years (PY) in 18-29, 30-39, 40-49, and 50-59 YOA (Table 1). Chronic disease HZ IR was higher than immunocompetent, but HZ case # 18-49 YOA was higher in immunocompetent than chronic disease. Immunocompetent HZ IR also increased with age: 0.77, 1.99, 2.35, and 3.24/1000 PY in the same groups. PHN % (Table 2) increased with age whereas hospitalization % (Table 3) was similar across same groups; both % were higher for chronic disease and overall than for same immunocompetent age groups. In the sensitivity analysis the IRs increased ∼30% on average.

**Table 3.**
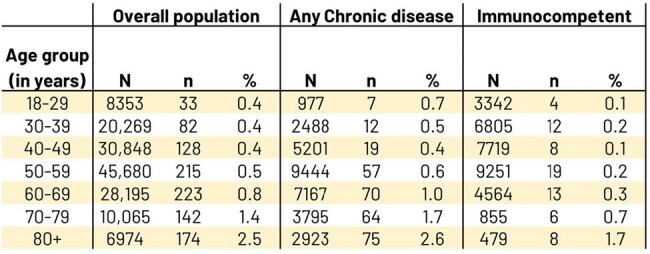
Number (%) of HZ-associated hospitalizations by age groups in: overall population, selected chronic disease, and immunocompetent participants

- N=number of people with HZ in age group.

- n=number of people from N who have HZ-associated hospitalization.

- %=n/N*100.

- Note: Age is computed at HZ event date.

**Conclusion:**

HZ burden increases with age among US ≥18 YOA across all populations, with a large number of HZ cases in immunocompetent 30-49 YOA without clear HZ risk factors. Although the incidence is high ≥50 YOA, the results highlight the burden in non-IC/AID 18-49 YOA.

Funding: GSK

**Disclosures:**

Rachel A. Cohen, MPH, GSK: Employed|GSK: Stocks/Bonds (Private Company) Yves Brabant, MEng, GSK: Employed Driss Oraichi, PhD, GSK: I am a full time employee of GSK|GSK: Stocks/Bonds (Private Company) Agnes Mwakingwe-Omar, MD, PhD, GSK: Employed|GSK: Stocks/Bonds (Private Company) Bruno Anspach, M.Mol.Bio, GSK: Employed|GSK: Stocks/Bonds (Private Company) Desmond Curran, PhD, GSK: employee|GSK: Stocks/Bonds (Public Company) Mitra Yousefi, PhD, GSK: Employed by GSK between March 2018 and November 2023|Moderna: Employee at Moderna since December 2023 Huifeng Yun, MD, PhD, GSK: fulltime employee of GSK|GSK: Stocks/Bonds (Public Company)

